# Association between prenatal exposure to perfluoroalkyl and polyfluoroalkyl substances and the incidence of infant and childhood respiratory infections: A systematic review

**DOI:** 10.3892/mi.2025.250

**Published:** 2025-06-24

**Authors:** Maria Lyrou, Grigorios Karampas, Dimitra Metallinou, Chrysoula Rozalia Athanasiadou, Kleanthi Gourounti, Vaidas Jotautis, Vasiliki Epameinondas Georgakopoulou, Demetrios A. Spandidos, Antigoni Sarantaki

**Affiliations:** 1 Department of Midwifery, School of Health and Care Sciences, University of West Attica, 12243 Athens, Greece; 2 Second Department of Obstetrics and Gynecology, Aretaieio Hospital, National and Kapodistrian University of Athens, 11528 Athens, Greece; 3 Faculty of Medicine, Kauno Kolegija Higher Education Institution, 50468 Kaunas, Lithuania; 4 Department of Pathophysiology, Laiko General Hospital, Medical School of National and Kapodistrian University of Athens, 11527 Athens, Greece; 5 Laboratory of Clinical Virology, School of Medicine, University of Crete, 71003 Heraklion, Greece

**Keywords:** perfluoroalkyl and polyfluoroalkyl substances, prenatal exposure, respiratory infections, childhood health, immunotoxicity

## Abstract

Perfluoroalkyl and polyfluoroalkyl substances (PFAS) are persistent environmental pollutants with potential immunotoxic effects. The present systematic review evaluated the association between prenatal PFAS exposure and the incidence of respiratory infections in infants and children. For this purpose, a comprehensive literature search was conducted across the PubMed, Scopus and Cochrane Library databases following the referred Reporting Items for Systematic Reviews and Meta-Analyses (PRISMA) guidelines. Studies assessing prenatal PFAS exposure and childhood respiratory infections were included. A total of six studies meeting the eligibility criteria were analyzed. Perfluorooctane sulfonate (PFOS) and perfluorooctanoic acid were the most frequently detected PFAS in maternal blood. While no significant associations were found in infancy, a greater prenatal exposure to perfluorohexane sulfonate and PFOS was linked to increased respiratory infections, particularly pneumonia and respiratory syncytial virus infections, in early childhood. Conversely, perfluorononanoic acid and perfluorodecanoic acid were associated with a reduced risk of lower respiratory tract infections and wheezing in children aged 4 to 7 years. The biological mechanisms likely involve PFAS-induced immune dysregulation, including altered cytokine responses and impaired vaccine effectiveness. The present systematic review highlights the complex role of prenatal PFAS exposure in modulating childhood respiratory health. Given the widespread presence of PFAS in the environment, regulatory policies should focus on minimizing exposure, particularly during pregnancy. Further research is required to clarify causal mechanisms and assess long-term respiratory outcomes in children.

## Introduction

Acute respiratory infections (ARIs) pose a significant burden on healthcare systems worldwide (1). According to epidemiological data, ARIs account for 20-40% of outpatient visits and 12-35% of hospital admissions in general healthcare settings (2). Pulmonary infections remain a leading cause of infant and childhood mortality, contributing to ~15% of all deaths among children <5 years of age globally (3,4). A previous cohort study estimated the incidence of ARIs at 1.8 episodes per infant per year, with upper respiratory infections comprising 95% of the cases (5).

The primary causes of ARIs in infants and young children include a variety of viral and bacterial pathogens. Viruses such as respiratory syncytial virus (RSV), influenza, rhinovirus, adenovirus, parainfluenza and human metapneumovirus are the most frequent causes of upper and lower respiratory tract infections in early life. Bacterial pathogens, including *Streptococcus pneumoniae*, *Haemophilus influenzae* and *Staphylococcus aureus*, are also implicated, particularly in more severe or secondary infections. The ability of the host to mount an effective immune response to these pathogens is essential in preventing complications. Consequently, any factor that compromises immune function during critical developmental windows, such as prenatal exposure to environmental immunotoxins, could potentially elevate the risk or severity of ARIs (6,7).

Perfluoroalkyl and polyfluoroalkyl substances (PFAS) constitute a group of synthetic chemicals extensively utilized in industrial and commercial applications since the mid-20th century. These compounds serve as surfactants and repellents in various products, including coatings for paper and packaging, textiles, leather, firefighting foams, photographic materials, cleaning products and pesticides (8-10). Due to their widespread use, PFAS have led to extensive environmental contamination on a global scale (10).

Human exposure to PFAS occurs through multiple pathways, including the ingestion of contaminated water and food. The highest concentrations have been detected in fish and shellfish, followed by red meat, animal fats, processed snacks (11,12) and beverages (13). Additionally, the inhalation and ingestion of dust represent key exposure routes (14). The bioaccumulation of PFAS in the bloodstream is directly proportional to the duration and level of exposure (15).

The persistence of PFAS in the environment, attributed to their chemical and thermal stability and high surface activity, along with their prolonged biological half-life, raises significant concerns regarding their potential impact on human health (8,15,16). Given their extensive use and resistance to degradation, PFAS have achieved global geographic distribution, with notable variations in exposure levels among different regions (15).

Furthermore, PFAS can cross the placental barrier and be transferred into breast milk, representing primary exposure sources for neonates (15). The fetal and early postnatal periods are critical windows for immune system development, increasing susceptibility to adverse effects from environmental exposures (15). Notably, prolonged breastfeeding has been associated with higher PFAS concentrations in infants, underscoring the need to elucidate the role of breastfeeding in the etiology of pediatric infectious diseases (15).

The European Food Safety Authority (EFSA) has recently established tolerable intake levels for a subset of PFAS based on their association with reduced antibody responses in children and adults, aiming to mitigate potential health risks (https://www.efsa.europa.eu/en/news/pfas-food-efsa-assesses-risks-and-sets-tolerable-intake). Despite increasing evidence of the toxicological effects of PFAS, data on their impact on infant and childhood respiratory health remain limited. Further research is essential to address this gap, particularly concerning the association between alternative PFAS compounds and respiratory infections in children, as research focuses on conventional PFAS (17).

Recent evidence has highlighted that PFAS exposure, particularly during critical periods such as gestation, may disrupt normal immune development (18). Several PFAS compounds have been shown to alter cytokine production, reduce antibody responses to vaccines, and impair immune cell function (18). Given that respiratory infections are largely managed through innate and adaptive immune responses, it is plausible that PFAS-induced immunotoxicity during fetal development could predispose children to developing more frequent or severe respiratory infections. Despite this biologically plausible link, only a limited number of studies have systematically investigated the association between prenatal PFAS exposure and respiratory health outcomes in early life (9-12).

The health implications of prenatal PFAS exposure are of growing concern, particularly given accumulating evidence linking PFAS to immunosuppression and altered vaccine responses in children. However, the respiratory consequences of *in utero* PFAS exposure remain inadequately characterized, despite respiratory infections being a leading cause of global childhood morbidity and mortality. The present systematic review addresses a critical gap by systematically evaluating whether prenatal PFAS exposure may increase susceptibility to respiratory infections during infancy and early childhood, a period marked by immune vulnerability. By clarifying these associations, the present study aimed to inform public health strategies and regulatory policies targeting maternal exposure to persistent environmental toxins.

## Data and methods

The present study was conducted according to the Preferred Reporting Items for Systematic Reviews and Meta-Analyses (PRISMA) guidelines (19). The present systematic review has been registered in the International Prospective Register of Systematic Reviews (PROSPERO) with ID no. CRD420251001057.

### Search strategy

A total of two independent reviewers conducted the literature search and screened the titles and abstracts for eligibility. Any disagreements were resolved through discussion or consultation with a third reviewer. A comprehensive literature search was conducted across three major electronic databases: PubMed/Medline, Scopus and the Cochrane Library. The search strategy was designed to identify relevant studies examining prenatal exposure to PFAS and their impact of the development of respiratory infections in children. The search algorithm incorporated a combination of controlled vocabulary (e.g., MeSH terms) and free-text keywords related to PFAS, including ‘perfluoroalkyl’ ‘polyfluoroalkyl’ ‘perfluorooctanoic acid (PFOA)’ ‘perfluorooctane sulfonate (PFOS)’ ‘perfluorononanoic acid (PFNA)’ ‘perfluorohexane sulfonate (PFHxS)’ and ‘perfluorobutane sulfonate (PFBS)’.

To ensure the identification of studies specifically addressing prenatal exposure, the search strategy incorporated terms such as ‘pregnancy’, ‘prenatal’, ‘gestational’, ‘maternal exposure’ and ‘maternal blood levels’. Boolean operators (AND, OR) were used to refine the search, and additional filters were applied where applicable. To enhance the comprehensiveness of the systematic review, reference lists of included studies were manually screened for additional relevant articles.

In addition to the primary database search, grey literature was considered to reduce potential publication bias, i.e., the tendency for studies with statistically significant or positive findings to be more likely published in indexed, peer-reviewed journals. Sources of grey literature included institutional repositories, governmental or non-governmental organization-funded cohort reports, conference proceedings, and preprint servers (e.g., medRxiv).

### Inclusion and exclusion criteria

Studies were included if they focused on pregnant women and women of reproductive age, assessed prenatal exposure to PFAS, and compared outcomes between exposed and non-exposed populations. The primary outcome of interest was the association between prenatal PFAS exposure and infant or childhood respiratory infections. Only primary research studies published in English were considered. The timeframe for inclusion was restricted to studies published between January 1, 2015, and February 1, 2025, and any studies conducted outside this period were excluded.

### The population, intervention, comparator, outcomes and study design (PICOS) framework

The PICOS framework for the present systematic review on prenatal exposure to PFAS and childhood respiratory infections can be outlined as follows: i) Population (P): Pregnant women and their offspring (infants and children); ii) intervention/exposure (I): Prenatal exposure to PFAS, including PFOS, PFOA, PFNA, PFHxS, perfluorodecanoic acid (PFDA), etc.; iii) comparison (C): Not applicable (no explicit comparison group in the included studies); iv) outcome (O): Incidence of respiratory infections in infancy and childhood, including pneumonia, RSV infections, bronchitis, and other respiratory tract infections; and v) study design (S): Observational studies (cohort, case-control, and cross-sectional studies).

### Outcomes

The primary outcome of interest in the present systematic review was the incidence of ARIs in infants and children associated with prenatal exposure to PFAS. ARIs included upper and lower respiratory tract infections, such as pneumonia, bronchitis, RSV infection, the common cold and streptococcal throat infections, as reported in the included studies.

Secondary outcomes included: i) The severity of respiratory infections, where available (e.g., number of infection episodes, presence of fever, co-occurrence of symptoms such as cough or nasal discharge); ii) lung function parameters [e.g., forced vital capacity (FVC), forced expiratory volume in one second (FEV_1_)]; c) Immune-related biomarkers, including alterations in immune cell subpopulations and cytokine profiles; and iv) hospitalization rates or clinical care utilization related to respiratory infections, when reported.

### PRISMA process

The initial search in the PubMed, Scopus and Cochrane Library databases yielded 458 records. Of these, 202 records were identified in PubMed/Medline, 253 in Scopus and three in the Cochrane Library. After removing 103 duplicate records, 355 unique records remained for further evaluation.

Each retrieved record underwent a detailed screening process to identify the most relevant studies. The first stage involved a review of titles and abstracts, eliminating studies that did not align with the research question, specifically those unrelated to the association between prenatal PFAS exposure and infant or childhood respiratory infections. Following this initial screening, 350 studies were excluded, leaving five studies eligible for full-text review.

**Figure 1:**
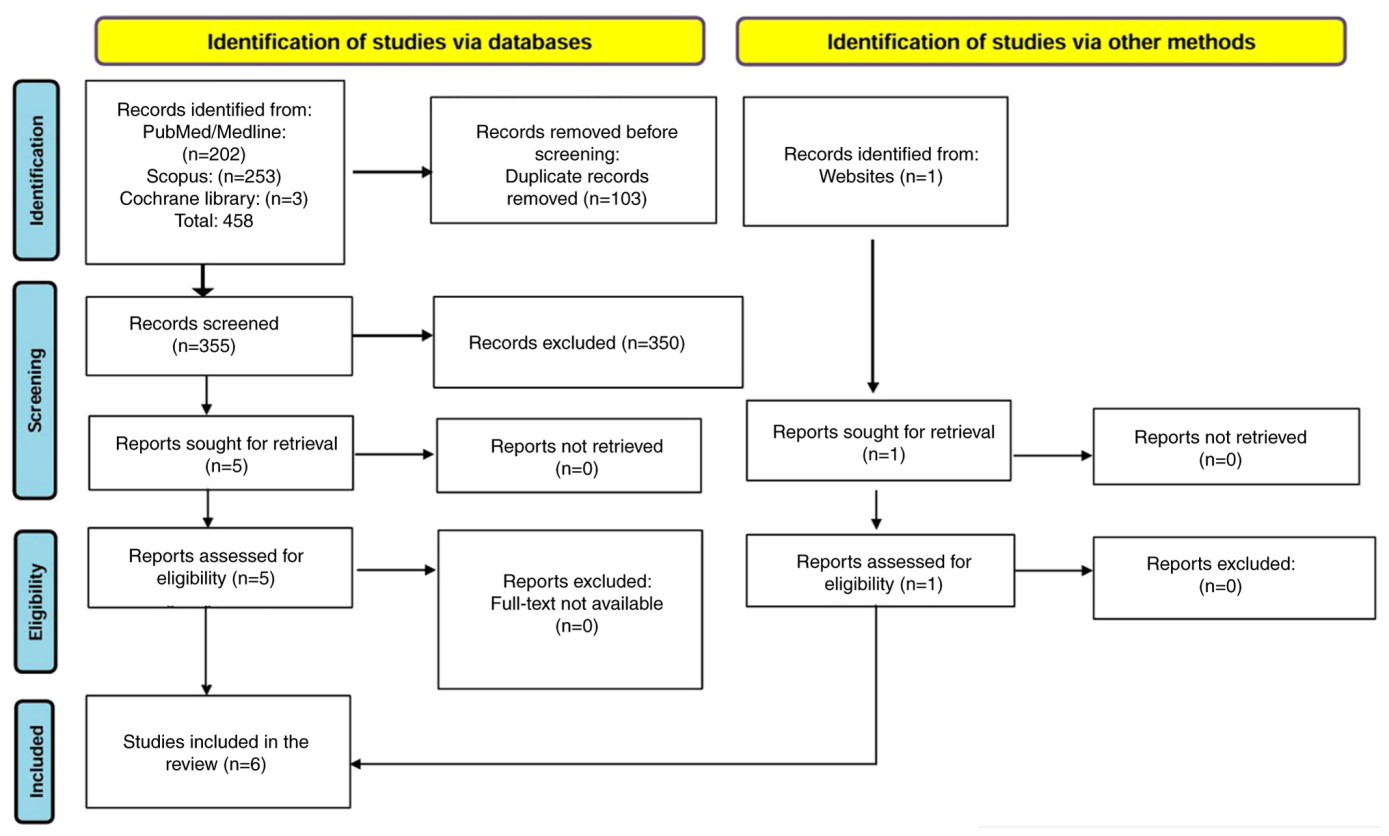
Flowchart for the study selection process.

All five studies were retrieved in full text and assessed for eligibility. Upon detailed examination, all five met the inclusion criteria and were retained for analysis. One additional relevant study, not retrieved from PubMed, Scopus, or the Cochrane Library, was identified through a manual search of reference lists and an institutional database. Although this record was not originally indexed in a peer-reviewed journal, it met tge inclusion criteria as a primary observational study with a defined methodology and sufficient data transparency. A final dataset of six studies were included in the present systematic review. The entire selection process is visually represented in Fig. 1, depicting the identification, screening, eligibility and final inclusion of studies.

### Quality assessment

The selected studies underwent a quality assessment based on the Caldwell framework, ensuring methodological rigor (20). The results of quality assessment are displayed in Data S1. The risk of bias and study quality were assessed independently by two reviewers. Any discrepancies in the quality assessment were discussed and resolved through consensus.

### Data extraction

Data extraction was performed independently by two reviewers using a standardized extraction form. Differences in data interpretation were addressed through discussion or resolved by a third reviewer if necessary. From the selected studies, several key variables were extracted for systematic analysis. These included the first author, year of publication, study design, sample size and data collection methods. The core findings of each study were examined, focusing particularly on their relevance to prenatal PFAS exposure and respiratory infections in infants and children. Additional factors, such as follow-up assessments, study limitations, and the country in which the research was conducted were also documented.

The findings of the selected studies were synthesized to provide a comprehensive understanding of the potential public health implications associated with early-life exposure to PFAS.

## Results

### Study selection

A total of six scientific studies were analyzed to investigate the association between PFAS and the occurrence of respiratory infections in infants and children (9-11,14,15,21). Among these studies, one study focused on infant infections, while the remaining five studies examined childhood infections.

### Study and patient characteristics

The six included studies were conducted in China, Denmark, Japan, Norway and Spain. All were prospective cohort studies focusing on mother-child pairs, with prenatal PFAS exposure assessed primarily through maternal blood samples collected during pregnancy. The majority of the studies employed questionnaires to gather outcome data, often supplemented by medical or birth records. Sample sizes varied considerably across studies. The smallest analytical cohort included just >230 mother-infant pairs, while the largest involved >1,500 such pairs. Some studies were nested within national birth cohorts, with original recruitment populations >6,000 pregnancies. Follow-up periods ranged from infancy to mid-childhood, extending up to 10 years in certain cases. Across the studies, measured outcomes included respiratory infections such as pneumonia, bronchitis, RSV, and throat infections, as well as fever episodes, lung function parameters, and general immune-related symptoms.

Sociodemographic characteristics, such as maternal age, education level and parity were often reported to influence PFAS levels, and several studies highlighted breastfeeding duration as a key determinant of ongoing postnatal exposure. However, differences in exposure assessment methods, follow-up duration and outcome definitions contributed to heterogeneity among studies. Each study was systematically reviewed, and key findings were extracted and organized in chronological order (Table I).

### PFAS exposure levels in maternal samples

An analysis of the selected studies confirmed the presence of PFAS in all maternal blood samples, with PFOS and PFOA being the most commonly detected compounds, followed by PFNA, PFDA, PFUA, PFHxS, perfluorododecanoic acid (PFDoA), PFBS, perfluorooctanesulfonamide (PFOSA), perfluoroheptanoic acid (PFHpA) and perfluoroundecanoic acid (PFUnDA) (15,21). Differences in PFAS levels were not observed in cases of infertility or pre-eclampsia (14). However, maternal parity, age and education level appeared to influence PFAS concentrations. Higher levels of PFOS, PFOA, PFHxS and PFNA were detected in nulliparous women compared to multiparous women (10). Additionally, PFAS concentrations, particularly PFOS and PFOA, were lower in older women and those with higher education levels (10). A higher pre-pregnancy body mass index was associated with lower PFDA concentrations, a pattern that was also observed for other PFAS (10). Another notable finding was the significant impact of breastfeeding duration on postnatal PFAS exposure, indicating that lactation may play a crucial role in ongoing exposure (14).

### Association between prenatal PFAS exposure and infant respiratory infections

In infants during the first year of life, no associations were identified between prenatal PFAS exposure and the occurrence of common colds, bronchitis or pneumonia (15). An inverse association between PFBS and these infections was initially suggested; however, further analysis did not support this finding (15). Additionally, no sex-based differences were observed in the association between PFAS exposure and acute infectious diseases in infancy (15). However, it was noted that a number of infants exhibited symptoms of infection within their first year, suggesting the need for additional research on potential contributing factors (10).

Beyond infancy, prenatal exposure to PFAS, particularly PFOS and PFHxS, was significantly associated with an increased risk of respiratory infections in early childhood. By the age of 4 years, pneumonia and RSV were among the most frequently reported infections, with similar prevalence in both sexes (P>0.05) (21). Notably, children in the highest quartile of PFOS exposure had a 61% increased risk of developing at least one infectious disease compared to those in the lowest quartile [odds, ratio (OR), 1.61; 95% confidence interval (CI), 1.18-2.21; P-value for trend=0.008). Furthermore, PFHxS exposure was positively associated with infectious diseases specifically among females, with the highest exposure quartile showing a 55% increased risk (OR, 1.55; 95% CI, 0.976-2.45; P-value for trend=0.045), whereas PFOS exposure was associated with infection risk in both sexes (21).

By the age of 2 years, PFUnDA was positively associated with the number of common cold episodes (11). By 3 years of age, PFOS and PFOA concentrations were inversely associated with the number of common colds, particularly among female children [risk ratio (RR) for PFOS, 0.94; 95% CI, 0.92-0.97; P<0.001; RR for PFOA, 0.96; 95% CI, 0.94-0.99; P=0.012] (14). However, bronchitis and pneumonia were significantly more frequent in children with higher prenatal exposure to PFOS, PFOA, PFHxS and PFHpS, with the effect of PFOA (RR, 1.27; 95% CI, 1.12-1.43) and PFHxS (RR, 1.15; 95% CI, 1.06-1.24) being stronger in girls (14). Streptococcal throat infections were positively associated with PFNA exposure (RR, 1.29; 95% CI, 1.11-1.50), while PFOA exhibited a stronger association in boys and PFUnDA in girls. Other throat infections were linked to PFHxS exposure in girls (RR, 1.10; 95% CI, 1.02-1.18) (14). Furthermore, children attending kindergarten at age three were more likely to develop pseudocroup, which exhibited a positive association with PFUnDA (inverse RR, 0.86; 95% CI, 0.78-0.95) in those attending childcare, highlighting a potential interaction between environmental exposures and social behaviors (14).

### Associations between PFAS exposure and fever in young children

Among children aged 1 to 4 years in the Odense Child Cohort, prenatal PFAS exposure was significantly associated with increased infection-related symptoms, independent of confounders such as smoking, breastfeeding duration, and sex (10). Specifically, children in the highest tertile of prenatal PFOS exposure experienced a 65% higher rate of fever days compared to those in the lowest tertile [incidence rate ratio (IRR), 1.65; 95% CI, 1.24-2.18; P<0.001], and the odds of having fever above the median level more than doubled (OR, 2.35; 95% CI, 1.34-4.11). A similar though slightly weaker association was observed for PFOA, with an increased odds ratio of 1.97 (95% CI, 1.07-3.62) for fever days above the median (10). Additionally, the co-occurrence of fever with cough or nasal discharge increased with higher PFOS and PFOA exposure. Notably, children in the medium PFOA exposure tertile had a 38% higher incidence of episodes with both fever and nasal discharge compared to the low-exposure group (IRR, 1.38; 95% CI, 1.03-1.86) (10). PFHxS also contributed modestly to these combined symptom patterns. Overall, these findings suggest that fever, as a sensitive and frequent symptom of infection (reported on average during 1.6% of days per child annually), may serve as a robust marker of increased infection susceptibility due to prenatal PFAS exposure (10).

### Association between PFAS exposure and respiratory health in children aged 4 to 7 years

In children aged 4 to 7 years, prenatal exposure to PFAS remained a key determinant of respiratory health outcomes. Higher concentrations of several PFAS were associated with a reduced risk of lower respiratory tract infections (LRTIs) and wheezing, with the exception of PFHxS, which was associated with an increased risk of LRTIs across all age groups (RR, 1.14; 95% CI, 1.00-1.29) (9). Higher levels of PFNA were linked to a lower probability of LRTIs at age 4 (OR, 0.85; 95% CI, 0.71-1.01) and wheezing at age 7 (OR, 0.69; 95% CI, 0.54-0.88). A protective association with asthma was also observed for PFNA (RR, 0.74; 95% CI, 0.57-0.96), while PFOS exposure was inversely associated with eczema (RR, 0.86; 95% CI, 0.75-0.98) (9).

However, stratified analysis by breastfeeding duration revealed an important effect modification: In children who were breastfed for <4 months, higher prenatal PFNA exposure was associated with an increased risk of asthma (RR, 2.73; 95% CI, 1.13-6.57). Furthermore, higher prenatal concentrations of PFOA were associated with lower lung function at age 4, as indicated by decreased z-scores in FVC (β, -0.17, 95% CI, -0.34 to -0.01) and FEV_1_ (β, -0.13; 95% CI. -0.29 to 0.03) These associations were not significant at age 7. No sex-based differences were observed in any of the respiratory outcomes assessed (9).

## Discussion

### Summary of key findings

The present systematic review synthesized evidence regarding the association between prenatal exposure to PFAS and the incidence of respiratory infections in infants and children. The findings highlight a complex interplay between PFAS exposure and respiratory health. They also revealed both positive and negative associations across different compounds and age groups.

The present systematic review of six studies confirmed the presence of PFAS in maternal blood samples, with PFOS and PFOA being the most detected compounds. Of note, four out of the six studies included in the present systematic review identified significant associations between prenatal PFAS exposure and an increased risk of respiratory infections in early childhood, with some evidence suggesting stronger associations in female children. These associations were particularly evident in female children. However, the findings were not entirely consistent across all age groups and PFAS compounds, suggesting that additional factors may modulate the observed associations.

### Interpretation by age group/outcome

In infants <1 year of age, no significant associations were detected between prenatal PFAS exposure and common respiratory infections such as colds, bronchitis, or pneumonia. However, by the age of 2 to 4 years, increased PFHxS and PFOS exposure was linked to higher susceptibility to respiratory infections, including pneumonia and RSV infections. These findings are consistent with experimental and epidemiological evidence outside the scope of the present systematic review, which indicate that PFAS may interfere with immune function and increase susceptibility to infections (22,23).

Notably, some PFAS compounds, including PFNA and PFDA, were found to be associated with a reduced risk of lower respiratory tract infections and wheezing, particularly in children aged 4 to 7 years. The observed inverse associations between PFNA and PFDA and certain respiratory outcomes, such as the reduced incidence of wheezing and lower respiratory tract infections, warrant cautious interpretation. These findings are counterintuitive given the known immunotoxic potential of PFAS and may reflect compound-specific immunomodulatory properties, differences in toxicokinetics, or residual confounding. For instance, PFNA and PFDA are longer-chain PFAS with different biological behaviors compared to PFOS and PFOA, potentially leading to differing impacts on immune cell differentiation or cytokine regulation. Additionally, these ‘protective’ associations may be influenced by factors, such as breastfeeding duration, socioeconomic status, or reverse causation, where healthier children are inadvertently more exposed due to differences in maternal behavior or environmental context. Given the small number of studies and inconsistent findings, further mechanistic and epidemiological studies are warranted to clarify whether these associations represent true protective effects or are the result of bias or uncontrolled confounding.

### Biological mechanisms

The biological mechanisms underlying the association between prenatal PFAS exposure and childhood respiratory infections remain incompletely understood. However, several hypotheses have been proposed. PFAS are known to interfere with immune function. They do so by altering cytokine production, reducing vaccine-induced antibody responses and impairing T-cell differentiation (22). These immunotoxic effects may increase vulnerability to infectious diseases during early childhood, a critical period for immune system development (23).

PFAS are considered to interfere with immune system development through several pathways. They may alter cytokine production, reduce antibody responses to vaccines and disrupt the balance of immune cell types. For example, prenatal exposure to PFOS, PFOA, PFNA and PFHxS has been shown to be associated with increases in certain immune cells called natural killer (NK) cells and a shift toward immune responses typically involved in allergies and inflammation (Th2 and Th17 pathways). At the same time, there appears to be a reduction in immune responses that fight viral and bacterial infections (Th1 responses). Some evidence also suggests that PFAS may weaken immune surveillance by lowering the number of cytotoxic T-cells involved in long-term protection and increasing markers of immune overactivation. These changes could help explain why higher PFAS exposure is linked to reduced vaccine effectiveness and greater vulnerability to infections in early childhood (22).

**Figure 2:**
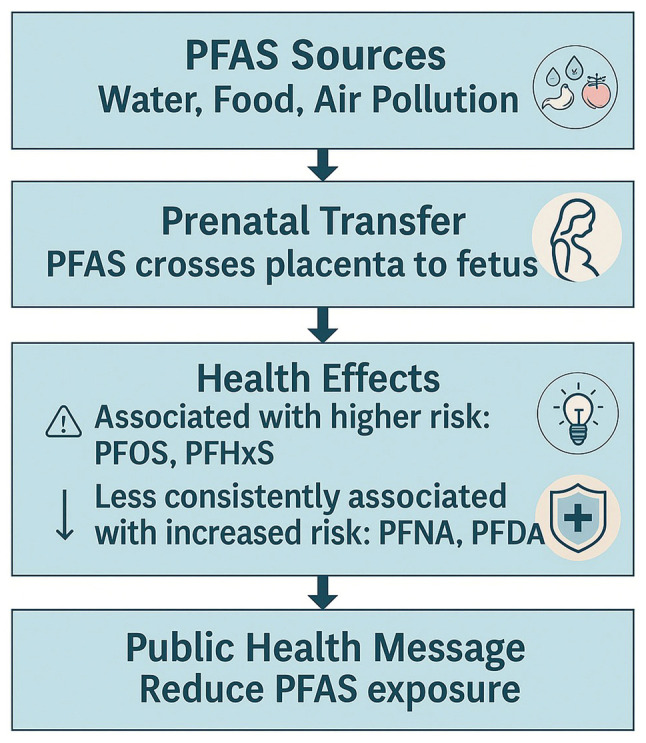
Pathway of prenatal PFAS exposure and its impact on childhood respiratory infections. PFAS, perfluoroalkyl and polyfluoroalkyl substances; PFOS, perfluorooctane sulfonate; PFOA, perfluorooctanoic acid; PFHxS, perfluorohexane sulfonate; PFNA, perfluorononanoic acid; PFDA, perfluorodecanoic acid.

**Figure 3:**
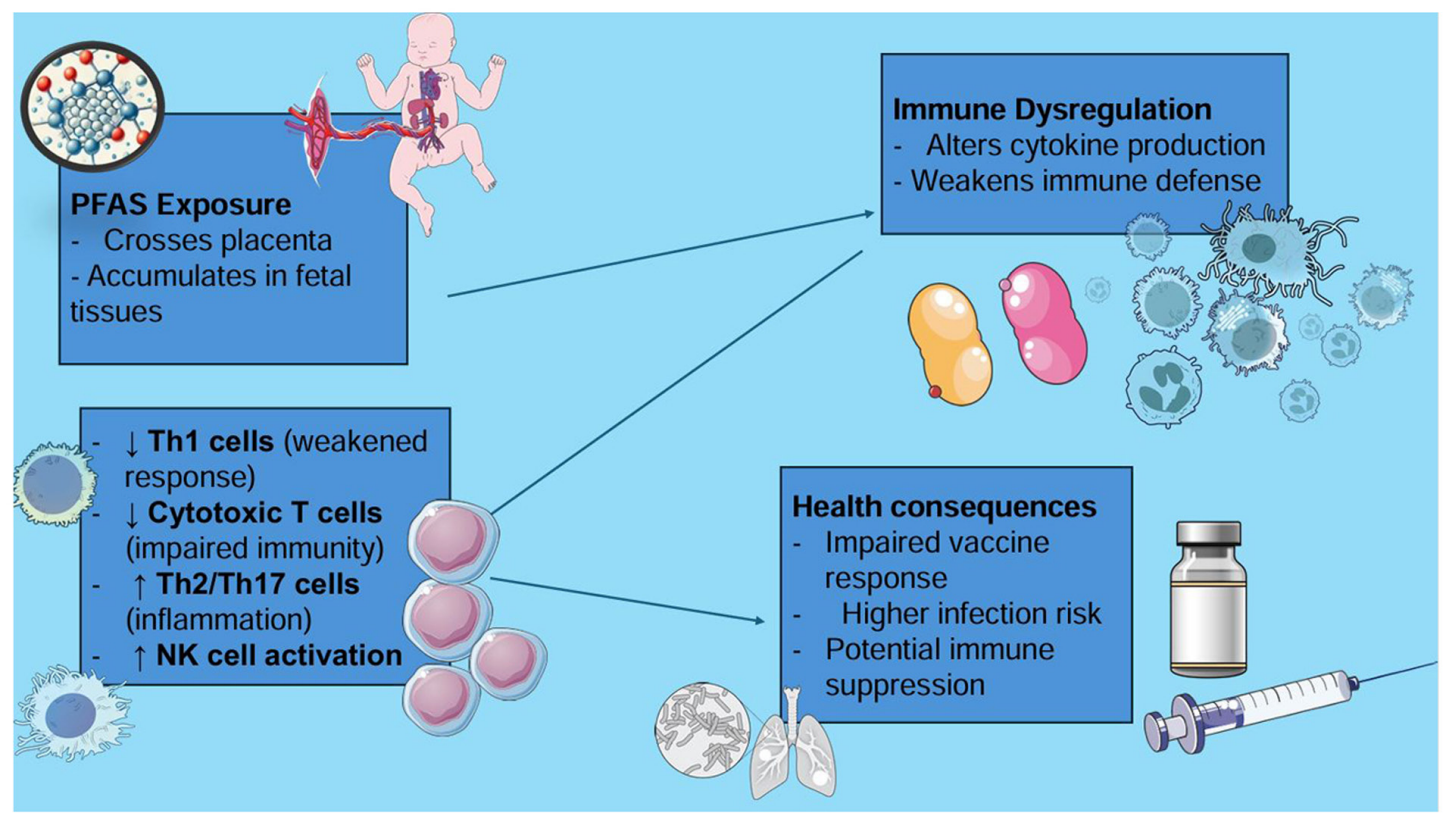
Impact of prenatal PFAS exposure on immune function and infection risk in childhood. PFAS, perfluoroalkyl and polyfluoroalkyl substances; Th1 cells, T helper 1 cells (involved in cellular immunity); Th2 cells, T helper 2 cells (associated with allergic responses); Th17 cells, T helper 17 cells (linked to inflammation and autoimmunity); NK cells, natural killer cells (involved in immune surveillance and viral defense). Parts of this image derived from the free medical site http://smart.servier.com/ (accessed on February 25, 2025) by Servier, licenced under a Creative Commons Attribution 4.0 Unported Licence.

Additionally, PFAS can cross the placental barrier and accumulate in fetal tissues. This may disrupt normal immune maturation. Their long biological half-life leads to ongoing postnatal exposure, especially through breastfeeding. A previous study highlighted the role of breastfeeding duration in modulating PFAS-related immune effects, with prolonged breastfeeding associated with higher PFAS concentrations in infants (15). This raises critical public health questions regarding the risks and benefits of breastfeeding in PFAS-contaminated environments. The proposed pathway linking prenatal PFAS exposure to childhood respiratory infections, highlighting key mechanisms, such as placental transfer, immune system modulation and the increased risk of infections is illustrated in Fig. 2. Furthermore, provides a visual representation of how prenatal PFAS exposure alters immune cell function, including shifts in Th1/Th2/Th17 balance and NK cell activity, contributing to increased infection risk in early life is provided in Fig. 3.

### Public health relevance

Given the widespread environmental contamination by PFAS, our findings have significant public health implications. Current regulations primarily focus on conventional PFAS compounds such as PFOA and PFOS, while emerging PFAS alternatives remain largely unregulated (24). Further research is required to assess the impact of these newer compounds on respiratory health outcomes. Moreover, public health interventions could consider strategies to reduce PFAS exposure during pregnancy and early childhood, especially in high-risk areas. Strategies such as regulating PFAS production and use, enhancing water filtration systems, and promoting dietary awareness may help mitigate potential health risks, although further evidence is needed to guide such measures (25).

### Limitations

While the present systematic review provides valuable insight into the association between prenatal PFAS exposure and respiratory infections, several limitations should be acknowledged. First, the included studies exhibit heterogeneity in study design, exposure assessment methods, and outcome definitions, which may contribute to inconsistency in findings. Second, the observational nature of the studies limits causal inferences, as residual confounding by socioeconomic status, environmental factors, and genetic predisposition cannot be ruled out. Third, potential publication bias may be present, given the limited number of eligible studies and the tendency for positive findings to be preferentially published in peer-reviewed journals. Although grey literature was included to mitigate this bias, the small sample size remains a constraint. Fourth, there was considerable variability in PFAS measurement approaches, including differences in the biological matrices used (e.g., maternal serum, cord blood), timing of sample collection, and analytical techniques, which may affect comparability of exposure estimates. Finally, ARIs were not uniformly defined across studies, and outcome assessment relied on varying clinical criteria or parental reports, which may introduce misclassification and reduce comparability.

### Critical appraisal of included studies

In evaluating the methodological quality of the six included studies using the Caldwell framework, several strengths and limitations were identified. All studies employed appropriate prospective cohort designs, and most recruited large and demographically diverse populations, enhancing generalizability. However, notable methodological weaknesses were observed. All studies relied heavily on self-reported outcomes, often obtained through parental questionnaires, introducing potential recall and misclassification bias. None of the studies explicitly described comparator groups, and only a few controlled for co-exposures or provided detailed adjustment for confounding factors such as environmental variables, socioeconomic status, or vaccination history.

The exposure assessment was generally based on maternal blood samples, but variation existed in the timing of sample collection, biomarkers used, and the analytical methods applied, affecting consistency across studies. Ethical approval and participant consent procedures were often assumed but not clearly stated. Only a few studies (9,14,21) combined self-reported outcomes with medical record validation, reducing the reliability of reported infections in most cases. Furthermore, substantial loss to follow-up was reported in at least two studies, potentially introducing attrition bias.

### Recommendations for future research

Future research should aim to address these limitations by conducting large-scale prospective cohort studies with standardized exposure and outcome assessments. Additionally, mechanistic studies are needed to elucidate the specific pathways through which PFAS influence immune function and respiratory health. Research should also explore potential sex-specific differences in PFAS susceptibility, as some studies suggest differential effects in males and females.

In conclusion, the present systematic review summarized current evidence on the association between prenatal exposure to PFAS and respiratory infections in infants and children. While several studies report associations between higher prenatal PFAS exposure, particularly to PFHxS and PFOS, and increased risk of respiratory infections in early childhood, findings are not entirely consistent across compounds or age groups. PFAS compounds, such as PFHxS and PFOS have been associated with an increased risk of respiratory infections, while others such as PFNA and PFDA may exert different immunomodulatory effects, which in some cases appear to correlate with reduced incidence of certain infections. These findings underscore the complex and potentially compound-specific nature of PFAS-related immune effects. However, the evidence remains limited by heterogeneity in study design, exposure assessment, and outcome definitions. Most studies are observational in nature, precluding causal inferences, and residual confounding cannot be excluded. Despite these limitations, the potential for PFAS to disrupt immune development during critical periods raises important concerns. Future research should prioritize longitudinal cohort studies with standardized exposure and outcome measures, as well as mechanistic investigations to clarify immunological pathways. From a public health perspective, reducing prenatal PFAS exposure through regulatory policies and environmental interventions may help mitigate potential risks, particularly among vulnerable populations such as pregnant women and young children.

## Supplementary Material

Caldwell Framework Quality Assessment for Included Studies (for the reference citations, please see the reference list in the main manuscript).

## Figures and Tables

**Table I:** Characteristics of the included studies.

Included studies						
Title	Association between prenatal exposure to perfluorinated compounds and symptoms of infections at age 1-4 years among 359 children in the Odense Child Cohort (10)	Prenatal exposure to perfluoroalkyl acids and prevalence of infectious diseases up to 4 years of age (21)	Prenatal exposure to perfluoralkyl substances (PFASs) associated with respiratory tract infections but not allergy- and asthma-related health outcomes in childhood (11)	Maternal levels of perfluoroalkyl substances (PFASs) during pregnancy and childhood allergy and asthma-related outcomes and infections in the Norwegian Mother and Child (MoBa) cohort (14)	Prenatal exposure to perfluoroalkyl substances, immune-related outcomes, and lung function in children from a Spanish birth cohort study (9)	Association between maternal serum concentration of perfluoroalkyl substances (PFASs) at delivery and acute infectious diseases in infancy (15)
Year of publication	2016	2017	2018	2019	2019	2022
Country	Denmark	Japan	Norway	Norway	Spain	China
Study type	Prospective cohort study	Prospective cohort study	Prospective cohort study	Cohort Study	Cohort study	Prospective study
Participants	6,707 pregnant women; 1,540 families	35,000 pregnant women	3754 healthy newborns with a birth weight of at least 2,000 g	2,000 mother-child pairs	2,150 pregnant women	773 pregnant women, >18 years old with singleton pregnancy
Focus group	649 samples, 359 mother-child pairs	1,558 mother-child pairs	641 infants	792 participants	1,214 mother-child pairs	235 mother-infant pairs
Measurement and data collection methods	Blood sample, questionnaires	Questionnaires, birth medical records, and maternal blood samples	Questionnaires	Questionnaires, maternal blood samples, and medical records	Questionnaires from interviews and maternal blood samples in the first trimester	Questionnaires, logistic and Poisson regression models
Measured outcome	Investigation of the association between prenatal exposure to PFAS and symptoms of infections at ages 1-4 years	Examination of the relationship between prenatal exposure to PFAA and the prevalence of infectious diseases up to 4 years of age	Determination of whether prenatal exposure to PFAS is associated with asthma, allergic diseases, or respiratory infections in childhood	Examination of the relationship between PFAS measured during pregnancy and childhood asthma, allergies, and common infectious diseases up to age 7	Association between prenatal exposure to PFAS and immune health, respiratory system, and lung function up to age 7	Investigation of associations between prenatal exposure to PFAS and acute infectious diseases in early childhood
Key findings	Positive association between prenatal exposure to PFOS and PFOA and the prevalence of fever, which may be a sensitive indicator of infection.	Prenatal exposure to PFAA may have immunotoxic effects on the immune system of offspring.	Prenatal exposure to PFAS was not associated with atopic or pulmonary conditions up to age 10, but some PFAS were linked to an increased number of respiratory infections in the first 10 years of life, suggesting immunosuppressive effects of PFAS.	i) Immunosuppressive effects of PFAS on respiratory infections such as bronchitis/pneumonia and throat infections, as well as diarrhea/gastric flu. ii) Prenatal exposure to PFAS was inversely associated with non-communicable urinary tract infections. iii) Possible role of sex in PFAS-health outcome associations.	i) Different PFAS may affect the developing immune and respiratory system differently. ii) Prenatal exposure to PFNA and PFOS may be linked to a reduced risk of respiratory and immune-related outcomes, while exposure to PFOA may be linked to reduced lung function in young children.	PFAS exposure was associated with an increased risk of diarrhea in the first year of life, with a higher effect among breastfed infants.
Follow-up	Questionnaires at 3 months, 18 months, and 3 years. Repeated text message every 2 weeks (26 times) over 1 year.	Three questionnaire completions (first trimester of pregnancy, 4 months postpartum, and 4 years postpartum).	At birth and every 6 months until age 2.	Data collected at 22 and 30 weeks of gestation and when the child was 0.5, 1.5, 3 and 7 years of age.	-	-
Study limitations	i) No information on PFAS exposure during childhood. ii) No data on fever treatment or vaccinations during the study period, potentially influencing fever duration. iii) Unmeasured confounding iv) Possibility of random findings.	i) Evaluation of infectious diseases based on maternal reports, not confirmed by medical records. ii) No studies on the validity of doctor-diagnosed infections. iii) No assessment of co-exposure to other environmental chemicals affecting the immune system. iv) Potential selection bias v) Postnatal PFAA levels and immune system biomarkers not examined.	i) Recall bias from questionnaires and interviews. ii) Lack of data on significant confounding factors.	i) Loss to follow-up. ii) Outcome dependence on questionnaires. iii) The number of infection episodes may be difficult for parents to recall and assess. iv) Uncertain counting of infection episodes. v) Significant loss to follow-up at age 7.	i) Use of maternal sample early in pregnancy as an indicator of fetal exposure. ii) Lack of information on PFAS exposure after birth. iii) Self-reported questionnaires for outcome assessment bias and misclassification. iv) The effect estimates of PFAS on lung function were small.	i) Measurement of maternal serum PFAS concentrations at delivery used as an indicator of prenatal exposure. ii) The validity of childhood infection studies is questioned due to misclassification of infectious disease causes. iii) Significant loss to follow-up or selection bias. iv) WQS regression cannot simultaneously determine associations in different directions for composite index elements. v) Potential influence of uncontrolled confounding factors.

MoBa, Norwegian Mother and Child Cohort Study; PFAS, perfluoroalkyl substances; PFAA, perfluoroalkyl acid; PFNA, perfluorononanoic acid; PFOA, perfluorooctanoic acid; PFOS, perfluorooctane sulfonate; WQS, weighted quantile sum.

## Data Availability

The data generated in the present study may be requested from the corresponding author.
