# Supplementary material for: Association between prenatal exposure to perfluoroalkyl and polyfluoroalkyl substances and the incidence of infant and childhood respiratory infections: A systematic review
Source: Med Int (Lond). 2025 Jun 24;5(5):51. doi: 10.3892/mi.2025.250 (PMC12209754; doi:10.3892/mi.2025.250)
Supplement: Caldwell Framework Quality Assessment for Included Studies (for the reference citations, please see the reference list in the main manuscript). [file Supplementary_Data.pdf]

Data S1

Caldwell Framework Quality Assessment for Included Studies (for the reference citations, please see the reference list in the main manuscript).

Study 1: Dalsager *et al*, 2016 (10)

1. Clear research question: Yes, the study investigates the association between prenatal exposure to PFAS and infection symptoms in early childhood.
2. Appropriate study design: Prospective cohort study, suitable for investigating exposure-outcome relationships over time.
3. Recruitment strategy: Large sample (6,707 pregnant women, 1,540 families), ensuring adequate representativeness.
4. Data collection methods: Blood samples and questionnaires, valid but with potential self-report bias.
5. Comparator group: Not explicitly defined.
6. Outcome measures: Infection symptoms, assessed through self-reported fever occurrence.
7. Statistical methods: Appropriate statistical methods used but limitations exist (potential confounders not measured).
8. Results and interpretation: Positive correlation between PFAS exposure and fever prevalence, but possible confounding and recall bias.
9. Ethical considerations: Ethical approval and informed consent not specified.
10. Limitations: No data on postnatal PFAS exposure, vaccination status, or fever treatment; potential unmeasured confounding and selection bias.

Study 2: Goudarzi *et al*, 2017 (21)

1. Clear research question: Yes, the study examines the association between prenatal PFAA exposure and childhood infectious diseases.
2. Appropriate study design: Prospective cohort study, enabling longitudinal assessment.
3. Recruitment strategy: Large cohort (35,000 pregnant women, 1,558 mother-child pairs), increasing generalizability.
4. Data collection methods: Blood samples, questionnaires, and medical birth records, but reliance on self-reports may introduce bias.
5. Comparator group: Not specified.
6. Outcome measures: Infectious diseases, reported by mothers, with no validation through medical records.
7. Statistical methods: Standard analytical techniques used, but no control for environmental co-exposures.
8. Results and interpretation: Indications of immunotoxicity from PFAA exposure, but findings are limited by self-reported outcomes.
9. Ethical considerations: Ethical approval assumed but not explicitly stated.
10. Limitations: No validation of reported infections, no consideration of postnatal PFAA exposure, potential selection bias.

Study 3: Impinen *et al*, 2018 (11)

1. Clear research question: Yes, assesses the impact of prenatal PFAS exposure on childhood asthma, allergies, and infections.
2. Appropriate study design: Prospective cohort study, appropriate for exposure-outcome assessment.
3. Recruitment strategy: 3,754 neonates included, ensuring a well-defined cohort.
4. Data collection methods: Questionnaires used; risk of recall bias exists.
5. Comparator group: Not explicitly stated.
6. Outcome measures: Asthma, allergies, respiratory infections, self-reported by parents.
7. Statistical methods: Analyses appear appropriate, but some confounders were not accounted for.
8. Results and interpretation: No link with atopic conditions, but PFAS exposure associated with increased respiratory infections.
9. Ethical considerations: Not explicitly mentioned.
10. Limitations: Recall bias from questionnaire data, lack of control for major confounders.

Study 4: Impinen *et al*, 2019 (14)

1. Clear research question: Yes, examines maternal PFAS levels and childhood health outcomes.
2. Appropriate study design: Cohort study, well-suited for longitudinal exposure-outcome analysis.
3. Recruitment strategy: 2,000 mother-child pairs, ensuring adequate representation.
4. Data Collection Methods: Blood samples, questionnaires, and medical records, improving reliability.
5. Comparator Group: Not explicitly stated.
6. Outcome measures: Childhood asthma, allergies, and infections, assessed via self-reports.
7. Statistical methods: Adequate, but issues exist in infection episode estimation.
8. Results and interpretation: PFAS exposure linked to respiratory infections and possible gender-based differences.
9. Ethical considerations: Not mentioned explicitly.
10. Limitations: Loss to follow-up, reliance on self-reported infections, possible outcome misclassification.

Study 5: Manzano-Salgado *et al*, 2019 (9)

1. Clear research question: Yes, investigates prenatal PFAS exposure and immune/respiratory outcomes in children.
2. Appropriate study design: Cohort study, allowing for long-term outcome tracking.
3. Recruitment strategy: 2,150 pregnant women, 1,214 mother-child pairs, ensuring statistical power.
4. Data collection methods: Blood samples and questionnaires, but self-reports introduce recall bias.

5. Comparator group: Not clearly defined.
6. Outcome measures: Immune and respiratory health, measured through self-reported symptoms.
7. Statistical methods: Proper analytical methods used, but effect sizes were small.
8. Results and interpretation: Mixed findings on PFAS effects, indicating different chemicals may have distinct impacts.
9. Ethical considerations: Not specified.
10 Limitations: Early maternal blood sample as exposure marker, lack of postnatal exposure data, recall bias in self-reported outcomes.

Study 6: Wang *et al*, 2022 (15)

1. Clear research question: Yes, assesses PFAS exposure and infectious diseases in infancy.
2. Appropriate study design: Prospective study, enabling exposure-outcome analysis.
3. Recruitment strategy: 773 pregnant women, 235 mother-infant pairs, relatively small sample size.
4. Data collection methods: Blood samples, questionnaires, and statistical modeling, with risk of misclassification bias.
5. Comparator group: Not stated.
6. Outcome measures: Acute infections, self-reported, with potential misclassification.
7. Statistical methods: Logistic and Poisson regression used, but confounders may not be fully controlled.
8. Results and interpretation: Increased risk of infant diarrhea with PFAS exposure, particularly in breastfed infants.
9. Ethical considerations: Not explicitly mentioned.
10. Limitations: Exposure measured only at birth, self-reported infections, substantial loss to follow-up, potential selection bias
